# Endoplasmic reticulum stress response in yeast and humans

**DOI:** 10.1042/BSR20140058

**Published:** 2014-07-01

**Authors:** Haoxi Wu, Benjamin S. H. Ng, Guillaume Thibault

**Affiliations:** *School of Biological Sciences, Nanyang Technological University, Singapore 637551, Singapore; †College of Life Sciences, Sichuan University, Chengdu 610064, China; ‡Lee Kong Chian School of Medicine, Nanyang Technological University, Singapore 308232, Singapore

**Keywords:** ER stress, IRE1, lipid disequilibrium, protein homoeostasis, unfolded protein response, UPR-related diseases. The UPR (unfolded protein response) is activated to alleviate the effects of ER (endoplasmic reticulum) stress and is highly conserved from yeast to human. In this review, we summarize the major advances in yeast and humans, AD, Alzheimer’s disease, ATF6, activating transcription factor 6, BiP, immunoglobulin heavy-chain-binding protein, bZIP, basic leucine zipper, CHOP, C/EBP homologous protein, cIAPs, cellular inhibitors of apoptosis proteins, cLD, core lumenal domain, eIF2α, eukaryotic initiation factor 2α, ER, endoplasmic reticulum, ERAD, ER-associated degradation, GADD34, growth-arrest and DNA-damage-inducible 34, GRP78, glucose-regulated protein 78, HD, Huntington’s disease, HSP70, heat-shock protein 70, Ire1, inositol-requiring enzyme-1, KEN, kinase-extension nuclease, MHC, major histocompatibility complex, NAFLD, non-alcoholic fatty liver disease, PD, Parkinson’s disease, PDI, protein disulphide isomerase, PERK, protein kinase-like ER kinase, PKR, protein kinase R, PS, presenilin, RIDD, regulated Ire1-dependent decay, *Sc*, *Saccharomyces cerevisiae*, *Sp*, *Schizosaccharomyces pombe*, UGT, UDP–glucose–glycoprotein glucosyltransferase, UPR, unfolded protein response

## Abstract

Stress pathways monitor intracellular systems and deploy a range of regulatory mechanisms in response to stress. One of the best-characterized pathways, the UPR (unfolded protein response), is an intracellular signal transduction pathway that monitors ER (endoplasmic reticulum) homoeostasis. Its activation is required to alleviate the effects of ER stress and is highly conserved from yeast to human. Although metazoans have three UPR outputs, yeast cells rely exclusively on the Ire1 (inositol-requiring enzyme-1) pathway, which is conserved in all Eukaryotes. In general, the UPR program activates hundreds of genes to alleviate ER stress but it can lead to apoptosis if the system fails to restore homoeostasis. In this review, we summarize the major advances in understanding the response to ER stress in *Sc* (*Saccharomyces cerevisiae*), *Sp* (*Schizosaccharomyces pombe*) and humans. The contribution of solved protein structures to a better understanding of the UPR pathway is discussed. Finally, we cover the interplay of ER stress in the development of diseases.

## INTRODUCTION

**Figure 1:**
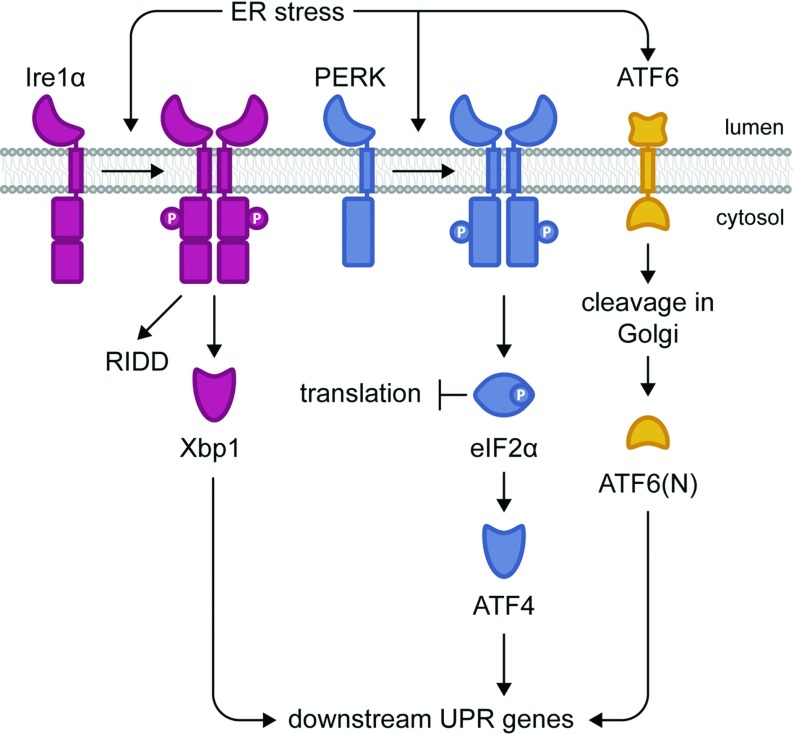
Activation of the UPR ER stress transducer, Ire1α, PERK and ATF6 form the three branches of the UPR pathways in mammals. In response to ER stress, the release of the molecular chaperone BiP, from the lumenal domain of Ire1α, promotes binding of misfolded proteins. Subsequently, Ire1α oligomerizes and phosphorylates itself, splices *XBP1* mRNA and this results in the translation of the transcription factor XBP1 regulating downstream signalling cascade. Upon prolonged ER stress, Ire1α cleaves mRNAs to relieve protein load through its RIDD activity. PERK oligomerizes and phosphorylates itself together with eIF2α, where it attenuates protein translation. It further activates the transcription factor ATF4, which carries out downstream activation of UPR genes. Under ER stress, ATF6 is packaged into vesicles and transported to the Golgi apparatus. Cleavage of ATF6 lumenal and transmembrane domain occur, where the N-terminal cytosolic fragment, ATF6(N) localize into the nucleus to activate UPR target genes.

Stress pathways respond to systemic perturbations by regulating diverse functions. They are specialized mechanisms designed to monitor and maintain intracellular homoeostasis. A response targeted to restore homoeostasis in the ER (endoplasmic reticulum), the UPR (unfolded protein response), is one of the best-studied cellular stress responses. Upon ER stress, three independent branches sense stress, with the Ire1 (inositol-requiring enzyme-1) branch being the most highly conserved among eukaryotes. In yeast, only the Ire1 pathway is found while metazoans utilize two additional pathways, double-stranded RNA-activated PERK (protein kinase-like ER kinase) and ATF6 (activating transcription factor 6) (Figure 1). In general, these ER-localized transmembrane proteins sense ER stress resulting in the activation of their respective pathways. Translational attenuation and activation of UPR target genes are the most common UPR outputs found in eukaryotes. However, the UPR programme can lead to apoptosis if cells fail to reach homoeostasis and undergo prolonged stress.

Early work on the UPR were done in animal cells, where the expression of ER-resident molecular chaperones [BiP (immunoglobulin heavy-chain-binding protein)/GRP78 (glucose-regulated protein 78), GRP94, PDI (protein disulphide isomerase)/ERp59 and ERp72] were shown to be induced by different treatments causing the accumulation of unfolded proteins in the ER [1,2]. The major breakthrough, reporting the missing elements in the UPR pathway from unfolded proteins to the activation of UPR-specific genes, was done in *Sc* (*Saccharomyces cerevisiae*). Upon accumulation of unfolded proteins, *HAC1* mRNA was found to be spliced by Ire1 protein, and only the spliced form results in translation of stable transcription factor Hac1 protein [3–5]. These findings opened the door to many new discoveries in yeast and metazoans. Although major advances in understanding the UPR come from all model organisms, this review will focus on the discoveries culminating from budding yeast, fission yeast and mammals, as well as diseases related to the UPR.

## ACTIVATION OF THE UPR IN YEAST

**Figure 2:**
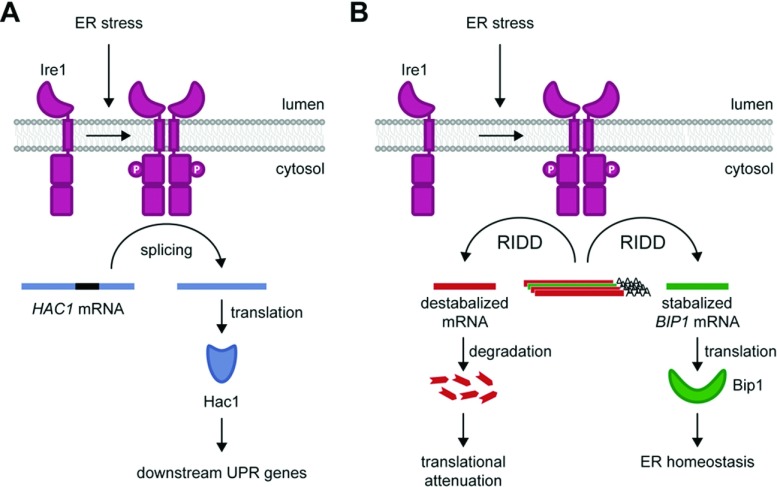
UPR in yeast (**A**) *S. cerevisiae* Ire1 is activated by ER stress. Upon activation, Ire1 undergoes *trans*-autophosphorylation and oligomerization. *HAC1* mRNA is spliced by activated Ire1 through its RNase domain. Upon translation, transcription factor Hac1 up-regulates UPR target genes to restore homoeostasis. (**B**) In *S. pombe* upon ER stress, Ire1 triggers downstream RIDD; the protein BiP1′s poly-A tail is recognized and cleaved by RIDD, but BiP1 protein is stabilized despite the cleavage of its mRNA. BiP1 protein is important for cell survival during stress condition; there are possible unknown candidates also involved in stress response, either in ER lumen or on ER membrane.

In *Sc*, ER stress is monitored by the transmembrane sensor protein Ire1 (Figure 2A). Ire1 is activated by either direct binding of unfolded proteins, or from the release of the molecular chaperone BiP from the lumenal domain of Ire1. Both mechanisms of activation have been proposed by different groups and will be discussed in detail in the Ire1 structure section. Upon activation, Ire1 oligomerizes followed by *trans*-autophosphorylation through its cytosolic kinase domain [6]. When activated, the cytosolic ribonuclease domain of Ire1 cleaves the intron of pre-messenger RNA *HAC1* to initiate synthesis of Hac1 transcription factor [3–5]. Hac1 then translocates into the nucleus to regulate the expression of UPR target genes. The UPR can alleviate stress by reversing severe dysfunctions through the up-regulation of nearly 400 target genes [7]. These target genes include ER chaperones, lipid biosynthesis enzymes and ERAD (ER-associated degradation) machinery. The UPR program appears to be adaptable and might be remodelled differently according to the needs of the cells. This differential regulation of the UPR, from different stressors, suggests the involvement of additional unidentified regulatory factors [7].

The UPR-related protein Ire1 is conserved in *Sp* (*Schizosaccharomyces pombe*) [8] (Figure 2B). Despite having no orthologues of *HAC1* or *XBP1* mRNA being identified, Ire1 still plays an important role to alleviate ER stress [9,10]. Unlike *Sc* Ire1, activated *Sp* Ire1 degrades ER-localized mRNAs to relieve protein load in a pathway called RIDD (regulated Ire1-dependent decay). This pathway was first identified in metazoan where Ire1 degrades mRNAs in addition to *XBP1* splicing [11,12]. Surprisingly, certain mRNAs cleaved by *Sp* Ire1 are stabilized instead of being degraded [10]. For example, *BIP1* mRNA, which encodes an HSP70 (heat-shock protein 70) family protein, is recognized and cleaved by Ire1, but it remains stable and its translation is increased. Notably, it was reported that a *BIP1* mRNA mutant, which is no longer cleaved by Ire1 in *Sp*, exhibits lower viability during ER stress. Other players are likely to work in synergy with Ire1 to regulate the UPR pathway. The UGT (UDP–glucose–glycoprotein glucosyltransferase) (*gpt1*) and a calnexin orthologues (*cnx1*) have been identified similarly with Ire1, from a genetic screen, to alleviate ER stress [9]. UGT was first identified from rat liver extract to recognize only misfolded proteins and to be essential under extreme ER stress in *Sp* [13,14]. The other identified protein from the screen, Cnx1, promotes folding of glycosylated proteins in the ER and may cooperate with BiP1 [15]. The first 160 residues of Cnx1 in *Sp* was found to be sufficient for cell survival under normal conditions [16,17]. Reinforcing its link to ER stress, *Sp* Cnx1 promotes apoptosis which is mediated by Ire1 [18]. Thus, the recent development in our understanding of ER stress response in *Sp* seems unique and it might help in elucidating the similar pattern in higher organisms.

## THE UPR ACTIVATION IN MAMMALS

In mammals, the presence of three different ER stress transducers facilitates the activation of the UPR [19] (Figure 1). Two forms of IRE1 are found in mammals where IRE1α is expressed ubiquitously while IRE1β solely in the intestinal and lung epithelium [20,21]. In human, IRE1α and IRE1β proteins are encoded by *ERN1* and *ERN2* genes, respectively. Ire1α branch of the UPR has been well studied because of its ubiquitous-expressing nature. The transcription factor downstream of Ire1α, XBP1, exhibits variance in its primary amino acid sequence with Hac1 but shares the common Ire1-mediated unconventional splicing activation of its mRNA and the bZIP (basic leucine zipper) motif. XBP1 activates similar downstream target genes as Hac1 in *Sc*, with the induction of genes involved in protein folding as well as in the secretory pathway [22]. With considerable redundancies existing between the three UPR pathways, IRE1–XBP1 pathway is dispensable to the activation of major chaperones such as BiP and GRP94 [23], but still plays crucial roles in ER homoeostasis and metabolic pathways [24].

PERK, which is an ER transmembrane kinase, mediates transcriptional and translational control of the UPR program [25]. Upon ER stress, PERK oligomerizes and phosphorylates itself together with eIF2α (eukaryotic initiation factor 2α) (Figure 1). eIF2α phosphorylation results in temporary attenuation of the overall protein translation and up-regulation of the transcription factor ATF4. This translation inhibition resultantly decreases the influx of proteins entering the ER, reducing ER protein folding load and alleviating ER stress. The absence of the PERK signalling cascade in *Sc* disallows the regulation of easing ER stress when protein integrity is compromised, where continued protein synthesis occurs even under ER stress. Paradoxically, certain mRNA are preferentially translated when eIF2α is limiting, one of which is transcription factor ATF4. Subsequently, ATF4 up-regulates CHOP (C/EBP homologous protein) and GADD34 (growth-arrest and DNA-damage-inducible 34). CHOP promotes ER stress-induced apoptosis [26] while GADD34 is involved in a negative feedback loop to counteract PERK by dephosphorylation of eIF2α, which resumes protein synthesis and sensitize cells to apoptosis [27]. Interesting, PERK also inhibits ER stress-induced apoptosis via induction of cIAPs (cellular inhibitors of apoptosis proteins) [28], and is a critical crosstalk regulator to influence the entire UPR in determining cell fate under ER stress [29]. In addition, another study has shown that activation of PERK could lead to down-regulation of anti-apoptosis protein XIAP, which could lead to increase in apoptosis [30]. These reports suggest that even when facing similar stress, cells could respond to it differently, and other factors might be involved to regulate how cells respond to various stresses, which could ultimately determine cell fate.

Upon detection of unfolded protein accumulation, ATF6 is packaged into vesicles and transported to the Golgi apparatus [31]. Cleavage of ATF6 lumenal and transmembrane domain occurs subsequently by S1P and S2P proteases, liberating the N-terminal cytosolic fragment, ATF6(N), for localization into the nucleus to activate UPR target genes [32]. A vast array of genes are activated downstream of ATF6(N), most noticeably BiP, PDI, and GRP94. Additional studies have shown that ATF6(N) is a major inducer for downstream response of ER chaperones and ERAD components [33,34].

Recent efforts to understand the involvement of factors participating in the UPR have shown little progress. Being intimately intertwined together, it would be hard to investigate the effect of individual factors, where compensation by other UPR branches could set in. The UPR has also been shown to be tightly linked to ERAD as well as lipid regulation [35], hence there is difficulty in isolating and understanding the UPR in cell physiology.

## UPR ACTIVATED FROM LIPIDS

**Figure 3:**
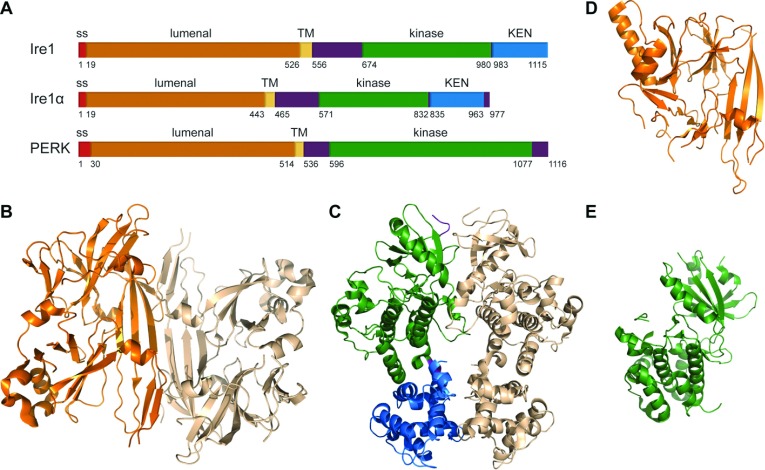
Solved protein structures of the UPR activation pathway (**A**) Schematic representation of yeast Ire1, human Ire1α and mouse PERK proteins. Ss (Signal sequence), TM (transmembrane domain), KEN domain. (**B**) Ribbons representation of dimerized cLD (amino acids 111–449) of *Sc* (*S. cerevisiae*) Ire1 [PDB (Protein Data Bank) code: 2BE1]. (**C**) Ribbons representation of the dimerized cytosolic domain of *Sc* Ire1 (PDB code: 3FBV). (**D**) Ribbons representation of human Ire1α luminal domain. The dimer adapts a back to back orientation (PDB code: 2HZ6). (**E**) Ribbons representation of PERK protein kinase domain from mouse (PDB code: 3QD2). All structures were drawn using *PyMOL* (www.pymol.org).

Many lipid synthesis genes are up-regulated from the UPR activation programme [36], indicating interconnection between lipid composition and ER stress. Inositol, which is an important regulator of lipid metabolism in yeast, is regulated by the UPR [37,38]. Moreover, deletion of fatty acid and sphingolipid biosynthesis genes such as *SUR4* and phospholipid synthesis genes such as *OPI3* or *INO1* activate the UPR [39,40]. The UPR can control lipid synthesis genes in order to balance membrane lipid composition through the IRE1–RIDD pathway [41]. Changes in membrane lipid composition may lead to the activation of the UPR. Previously, we demonstrated that the UPR remodels protein homoeostasis network, in yeast cells, instead of restoring lipid composition under global lipid disequilibrium [40]. Lipid imbalance also contributes to the disruption of calcium homoeostasis in mammals [42]. Other than causing accumulation of misfolded proteins, calcium metabolic imbalance results in the accumulation of free fatty acids causing the activation of the UPR [43]. Alternately, membrane lipid composition imbalance can be directly sensed by Ire1 protein resulting in UPR activation. An *Sc* Ire1 mutant failing to bind unfolded proteins, bZIP Ire1, was observed to activate the UPR normally during lipid imbalance [44]. This study suggested, for the first time, that Ire1 can sense stress from its transmembrane or cytosolic domains. More recently, a novel mechanism of UPR activation, during lipid perturbation, was proposed in mammalian cells [45]. Ire1α and PERK proteins lacking their luminal domain sense saturated lipids and they are sufficient to activate the UPR in mammalian cells (Figure 3A). Both activators undergo phosphorylation, and activate their downstream pathways similarly to their respective full-length protein. In addition, PERK was shown to directly sense exogenous saturated fatty acid by *in vitro* liposome reconstitution [45]. Thus, lipid and protein metabolisms are tightly regulated and share common regulatory pathways.

## IRE1 STRUCTURE AND ACTIVATION

To better understand the pathways leading to UPR activation, substantial efforts have been made to solve protein structures, in particular the ER stress transducers. However, due to the variable hydrophobicity within membrane proteins and the difficulty to solubilize them, it has been challenging to obtain structures at high resolution [46]. The first reported structure of UPR transactivator was the cLD (core lumenal domain, amino acids 111–449) of Ire1, from *Sc* at 2.98 Å resolution [47] (Figure 3B). cLD dimer exhibits a groove similar to MHC (major histocompatibility complex). From this observation, it was proposed that the direct binding of unfolded proteins to Ire1 lumenal domain is sufficient to activate the UPR in yeast.

Before acquisition of the Ire1 lumenal domain structure in *Sc*, it was accepted that Ire1 is activated by the release of Hsp70 molecular chaperone BiP from its lumenal domain. The importance of ER-resident Hsp70 family proteins, during stress conditions, was reported by several groups where they are up-regulated under ER stress [1,48,49]. Moreover, overexpression of BiP is sufficient to attenuate the UPR to stress [50]. In *Sc*, BiP was reported to serve as a negative regulator of the UPR [51]. BiP keeps Ire1 inactive by directly binding its lumenal domain but during ER stress, it dissociates to bind unfolded proteins resulting in Ire1 activation.

However, several other studies contradict this model and support the direct binding model. An *Sc* Ire1 lumenal domain deletion mutant was generated to disrupt BiP binding [52]. In this mutant, the UPR activation level appeared normal during ER stress. It indicates that BiP Ire1 interaction is not required to activate Ire1. Thus, the dissociation of BiP and self-association of Ire1 is not sufficient to activate the UPR suggesting that more steps are involved in the activation pathway [53]. Interestingly, several studies reported that Ire1 directly binds unfolded proteins and prevent protein aggregate formation [44,54–56]. In addition, Ire1 preferentially binds to peptides enriched in basic and hydrophobic residues reinforcing its affinity for misfolded proteins (Gardner and Walter [54], #23). The binding pattern was proposed to be like ligand–receptor interaction in which BiP serves by preventing Ire1 oligomerization [49].

The cytosolic domain of Ire1 has two functional domains with kinase and RNase enzymatic activities [57] (Figure 3A). Structural studies of Ire1 cytosolic domain helped in dissecting the cascade of events of the UPR activation. The first crystal structure of Ire1 cytosolic domain was at 2.4 Å resolution [58] (Figure 3C). Based on its motif folding, RNase domain was redefined as the KEN (kinase-extension nuclease) domain. KEN domain possesses ribonuclease activity after *trans*-autophosphorylation of its adjacent kinase domain. Interestingly, there is no known protein other than Ire1 with both kinase and RNase activities. Ire1 KEN domain is similar to mammalian RNase L, which cleaves endogenous and viral RNA within the cell upon activation from interferon [59,60]. Four key residues (Y1043, R1056, N1057 and H1061) of Ire1 form the RNase active site and they are conserved in RNase L [58]. The oligomerization of Ire1 cytosolic domain is essential to its enzymatic activity, which is driven by the oligomerization of Ire1 lumenal domain [61]. Together with the structural information of Ire1′s RNase inactive mutant [62], it was revealed that the kinase activity of Ire1 is not required during the UPR activation cascade but essential in Ire1 deactivation [63]. On the contrary, RNase activity is essential for the same process.

In both yeast and human, dimerized Ire1 form MHC-like grooves [64]. However, the Ire1α groove is thought to be too small to accommodate unfolded proteins (Figure 3D). Gln105 of Ire1α, which forms hydrogen bond to adjacent residues, prevents direct binding of unfolded proteins. Thus, it supports the conventional indirect UPR activation model. In this model, BiP dissociates from Ire1α and triggers its oligomerization and phosphorylation. After activation, Ire1α adopts a face-to-face orientation when its phosphorylation is prevented with ATP competitor sunitinib [65]. In contrast, dimerized *Sc* Ire1 RNase domain holds a back-to-back orientation when activated. As a result, face-to-face orientation of Ire1α would compromise the activation of the UPR by failing to splice *XBP1*. The different activation mechanism of Ire1α lumenal domain, compared with *Sc* Ire1, might confer greater specificity in responding to ER stress and thus complementing the other two branches of the UPR. It is worth mentioning that there is still no full-length structure of Ire1 reported yet. Structural information of Ire1 transmembrane domain would be valuable to give us a better understanding of its role in sensing lipid perturbation [44,45].

## PERK AND ATF6 STRUCTURES

Recently, the structure of PERK's kinase domain was solved at 2.8 Å resolution [66] (Figure 3E). Upon activation of PERK, its cytosolic kinase domain adapts a back-to-back dimer structure, and its dimerization is driven by PERK ER lumen domain dimerization. This further activates PERK's kinase domain resulting in *trans*-autophosphorylation [57]. *Trans*-autophosphorylation of PERK activation loop (residues 953–990) is indispensable for its activity [66]. PERK kinase domain shares sequence similarity with PKR (protein kinase R). It was reported before that PKR phosphorylates eIF2α at Ser^51^, leading to inhibition of translation [67]. Thus, it is not surprising that activated PERK also phosphorylates eIF2α to alleviate ER stress [66].

The 3D structure of ATF6 remains to be elucidated. ATF6 is synthesized as a precursor protein (p90ATF6), which is localized at the ER in unstressed cells. In response to ER stress, ATF6 is packaged into vesicles and transported to the Golgi apparatus and the N-terminal fragment is cleaved off (p50ATF6) [31,32].

## UPR AND DISEASES

**Figure 4:**
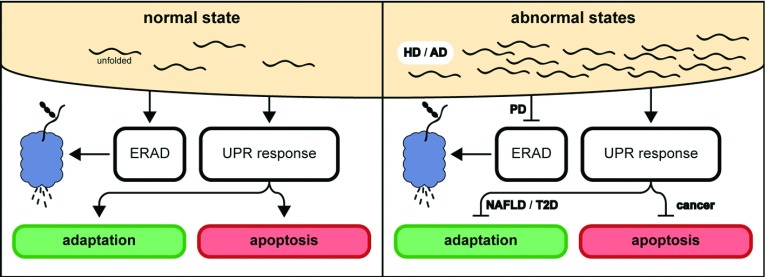
Diseases linked to ER stress During ER stress, misfolded proteins arising in the ER is assisted with chaperones for refolding, and failure to be refolded to their native state would result in their degradation via the ERAD pathway. The UPR is activated with the accumulation of unfolded or misfolded proteins, which would then halt protein translation and induce stress-response genes. Under prolonged ER stress, apoptosis is initiated by the UPR. Diseased states often arise from the failure of the UPR to respond well under ER stress, or from an accumulation of unfolded proteins. Inadequate response of the UPR could result when elements in the UPR signalling cascade is down-regulated and hence, a sufficient response could not be mounted to alleviate ER stress. Diseases such as NAFLD, T2D (type II diabetes) and cancer are implicated in this model. Mutations in protein coding genes could cause proteins synthesized to be misfolded and form aggregates rapidly. This could be severe that the ERAD fails to degrade the proteins adequately and the UPR is unable to compensate for the ER stress. This model often includes degenerative diseases such as PD, HD and AD.

Proteins are the key regulators of cellular diversity in function, performing numerous roles such as enzymatic reactions, mechanical support, transport of substrates and signal transduction. The UPR maintains the integrity of protein synthesis and alleviates ER stress through the regulation of various proteins. Hence, UPR signalling is involved deeply in numerous physiological processes besides protein quality control [68]. Several diseases such as diabetes, NAFLD (non-alcoholic fatty liver disease), cystic fibrosis, PD (Parkinson's disease), HD (Huntington's disease), AD (Alzheimer's disease), inflammation, cancer and liver failure are associated with the UPR pathways [69] (Figure 4). Implications of the UPR in metabolic diseases, cancer and neurodegenerative diseases will be reviewed in this section.

NAFLD is an emerging and now the most common cause of chronic liver enzyme elevations and cryptogenic cirrhosis because of the prevalence of obesity [70]. The failure of the UPR to rapidly re-establish ER homoeostasis via genetic ablation of *eIF2α*, *IRE1α* or *ATF6α* results in hepatic steatosis, where the capacity to oxidize fatty acids is impaired [71]. This is further exacerbated by dysfunction in the lipoprotein secretion pathway. It was reported in another study that heterozygous ATF4 mice benefitted in protection from diet-induced obesity and diet-induced hepatic steatosis [72]. Overexpression of mediators involved in UPR such as *GRP78* improves insulin action and hepatic steatosis [73]. Other metabolic diseases such as type 2 diabetes could also be the consequence of ER dysregulation. ER stressor deficiency in XBP1 induces the development of insulin resistance [24], where body cells fail to respond to insulin in uptaking glucose. In other studies to reveal the mechanism of how ER stress develops in obesity, the authors have found that free fatty acids could be the trigger of ER stress via perturbation of ER membrane integrity [74,75]. Increased mTOR (mammalian target of rapamycin) signalling pathway, which regulates cell proliferation and survival, is also found in most obese patients to block insulin signalling pathways [76]. Additionally, disruption of signalling between p85s and XBP1 occurs in obesity, where p85 serves to activate XBP1 after insulin simulation, resulting in decreased localization of XBP1 to the nucleus for the activation of the UPR [77]. Thus, the UPR appears deeply intertwined with lipid homoeostasis, where dysregulation in the UPR is often manifested in various metabolic diseases.

Neurodegenerative diseases frequently arose from the accumulation of misfolded proteins, leading to the death and loss of neurons necessary for physiological functions [78,79]. In PD, the *Parkin* gene which encodes an E3 ubiquitin ligase is mutated and results in the failure of substrate degradation. This subsequently leads to unfolded protein accumulation, causing UPR activation and eventually apoptosis of neurons through the action of CHOP [80,81]. The accumulation of abnormally long huntingtin protein from the increase in CAG nucleotide repeats in the *huntingtin* gene results in HD [82]. Accumulation of aggregates impairs the proteasome degradation system, contributing to the accumulation of other misfolded proteins and subsequently triggering ER stress [83]. Other evidence such as elevated expression of UPR target genes; *CHOP*, *GRP78* and *Herp* are found in patients with HD [84], and perturbation of ER calcium homoeostasis is linked to a mutant *huntingtin* gene [85]. This suggests that the mutant *huntingtin* continuously activates UPR target genes and have wide spread consequences on other facets of ER homoeostasis. AD is characterized by aggregation of fibrous insoluble proteins, commonly the amyloid-β peptide [86]. Accumulation of unfolded proteins, elevated ER stress, and activation of the UPR are commonly found in many cases of patients with AD [87,88], compelling a model where there is accumulation of misfolded proteins instead of dysregulation of the UPR. PS (presenilin) are highly conserved transmembrane proteins which regulates the cleavage of other proteins at their transmembrane domain, and is involved in ER calcium trafficking and homoeostasis [89]. They are revealed to have as many as 100 mutation variations in familial AD, signifying the importance of PS in its contribution to AD. Neurons are particularly sensitive to toxic aggregate-prone proteins, and hence neurodegenerative disease often arises from the buildup of such protein plaques. The accumulation of aggregates in the cytosol could indicate similar approaches in therapeutic treatment, and further understanding of the involvement of the UPR in the development of the disease could provide clues.

The involvement of the UPR in tumourigenesis is intimate yet complex. Following the onset of malignancy and rapid tumour growth, inadequate vascularization could subsequently result in microenvironmental stress such as hypoxia and depletion of nutrients. Additionally, tumour-intrinsic stress factors such as defects in synthesis of biomolecules arising from mutations in the genes could elevate ER stress further [90,91]. The paradox of the UPR in tumourigenesis begins, where UPR up-regulates protein folding capacity and ensures the continued integrity of protein folding, upholding cell survival [92]. The UPR has been shown to contribute in this fashion to protect tumorigenic cells from undergoing apoptosis under hypoxia conditions [93]. GRP78 is found as a critical factor in promoting cancer; increasing proliferation, avoiding apoptosis and the promotion of angiogenesis [94]. However, when chronic ER stress sets in, failure to restore ER homoeostasis causes the UPR to activate the apoptotic cascade in tumour cells. CHOP is a key regulator [95] but not the sole factor [26] involved in inducing apoptosis, in which the alteration of several genes by CHOP leads to activation of various apoptotic pathways [92,96].

## CONCLUSION AND PERSPECTIVE

The recognition that the UPR is deeply associated with various diseases, especially in metabolic and neurodegenerative diseases, allows us to appreciate the UPR pathways better. However, interactions between the three branches are not well characterized, and more has to be done in order to elucidate a more detailed mechanistic understanding of the UPR elements. This would allow for the effective manipulation of factors involved in the UPR, which could prove extremely valuable in developing therapeutic treatments for such diseases. While there is no universal treatment, it will be useful to draw parallel approaches to treatment when diseases are found to share conspicuous characteristics or causes. Identifying and targeting the UPR elements, which fail to perform before the development of chronic ER stress would be ideal. Induction of such UPR factors naturally or artificially via exogenous means could prevent the progression of UPR-related diseases. In less preferred but still beneficial cases, artificial means such as chemical chaperones [76] could ease mild ER stress or prolong the onset of diseases. Alleviating ER stress via increasing ER-folding capacity could be beneficial for obesity or cystic fibrosis [76] by increasing insulin sensitivity or reducing the secretion of mutated proteins. In other circumstances, targeting the UPR to promote the apoptotic cascade would be ideal for diseases such as in cancer and TSC (tuberous sclerosis complex) [92,97]. Novel methodological approaches in regulating or influencing the UPR would allow greater flexibility and sensitivity to be applied to various diseases implicated by the UPR.
